# Peak Flow as a Predictor: Identifying When Surgical Intervention Is Required in Recurrent Subglottic Stenosis

**DOI:** 10.7759/cureus.83476

**Published:** 2025-05-04

**Authors:** Ronak S Patel, Daniel Sherlock, Mae Leef, Abdul H Alraiyes

**Affiliations:** 1 Department of Internal Medicine, Advocate Lutheran General Hospital, Park Ridge, USA; 2 Department of Interventional Pulmonology, Advocate Lutheran General Hospital, Park Ridge, USA

**Keywords:** balloon dilation, idiopathic subglottic stenosis, peak expiratory flow, recurrent subglottic stenosis, remote monitoring

## Abstract

Subglottic stenosis (SGS) is a narrowing of the airway below the vocal cords, caused by congenital, idiopathic, or acquired factors. Management of SGS is difficult due to high recurrence rates. Objective tools, such as the SGS-6 questionnaire, peak expiratory flow (PEF), and expiratory disproportion index, have shown promise in assessing disease severity and guiding treatment timing. This case presents a 64-year-old man whose SGS was successfully managed using home PEF meter readings to guide timing for bronchoscopic intervention with balloon dilation. Remote tools, such as PEF monitoring, have proven effective in identifying the need for timely intervention. This case report aims to highlight the role of PEF in monitoring SGS progression and predicting the need for the next bronchoscopic intervention. Further validation of these remote tools should be encouraged to enhance patient outcomes, track disease progression, and streamline clinical workflows.

## Introduction

Subglottic stenosis (SGS) is a condition characterized by the narrowing of the airway below the vocal cords and above the trachea [1]. The etiology of SGS can be idiopathic, congenital, or acquired [1]. Acquired SGS is often related to laryngeal trauma, fibrosis from endotracheal intubation, malignancy, thermal injuries, or autoimmune processes [1]. Patients commonly present with stridor, dyspnea, and hoarseness.

Management involves various treatment modalities, including antireflux medications, steroid injections, and bronchoscopic dilations [1]. In severe cases, tracheostomy or tracheal resection with anastomosis may be required [2]. Although patients often experience symptom improvement following intervention, recurrence rates remain high, making SGS a challenging condition to manage [2]. This places a significant burden on patients, who may require frequent follow-ups and serial airway evaluations to monitor postintervention outcomes.

We present a case in which idiopathic SGS was successfully managed using close monitoring of home peak expiratory flow (PEF) readings to guide decision-making and optimize the timing of bronchoscopic intervention.

## Case presentation

The patient is a 64-year-old man with a past medical history of gastroesophageal reflux disease and SGS requiring multiple prior subglottic dilations. He presented to the hospital with worsening respiratory symptoms, including stridor, shortness of breath, wheezing, and difficulty clearing secretions with coughing. After ruling out an infectious etiology, it was concluded that the patient was experiencing a recurrence of SGS, given his history of SGS and multiple prior presentations with nearly identical symptoms.

**Figure 1:**
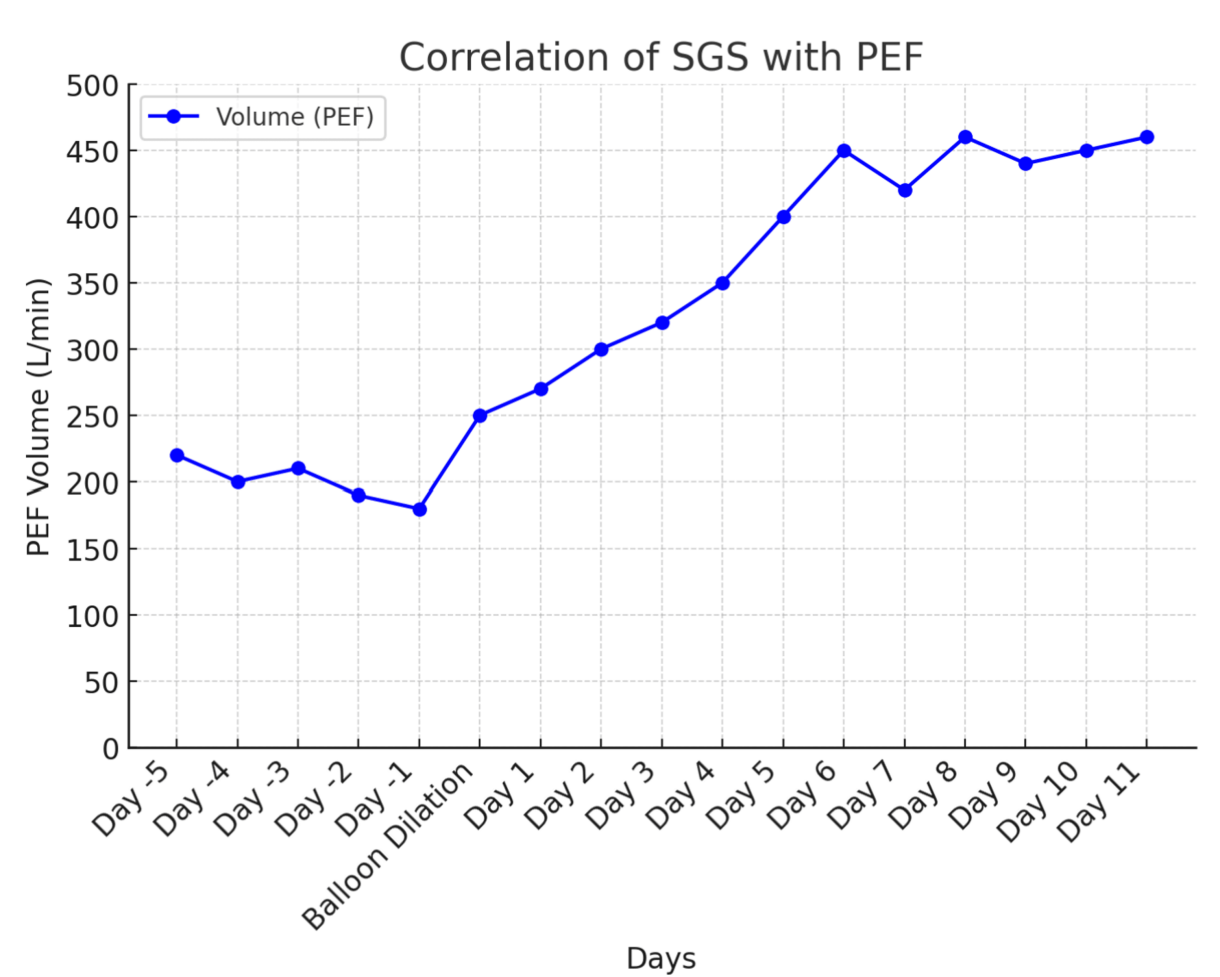
Graph portraying the patient’s self-recorded home PEF values both before and after balloon dilation SGS: subglottic stenosis; PEF: peak expiratory flow

Following the patient’s initial subglottic dilation, he was advised to monitor his PEF at home once daily using a peak flow meter and record these values in a log. As his symptoms progressed, initially with worsening cough and wheezing, and later with shortness of breath and audible stridor, he noted a corresponding decline in his PEF values. His baseline PEF values range around 400 L/minute. At the time of presentation, his PEF had decreased to 250 L/minute, indicating a 37.5% drop from his baseline. This was a level that, based on previous episodes, aligned with the threshold at which he had previously required dilation (Figure 1).

**Figure 2:**
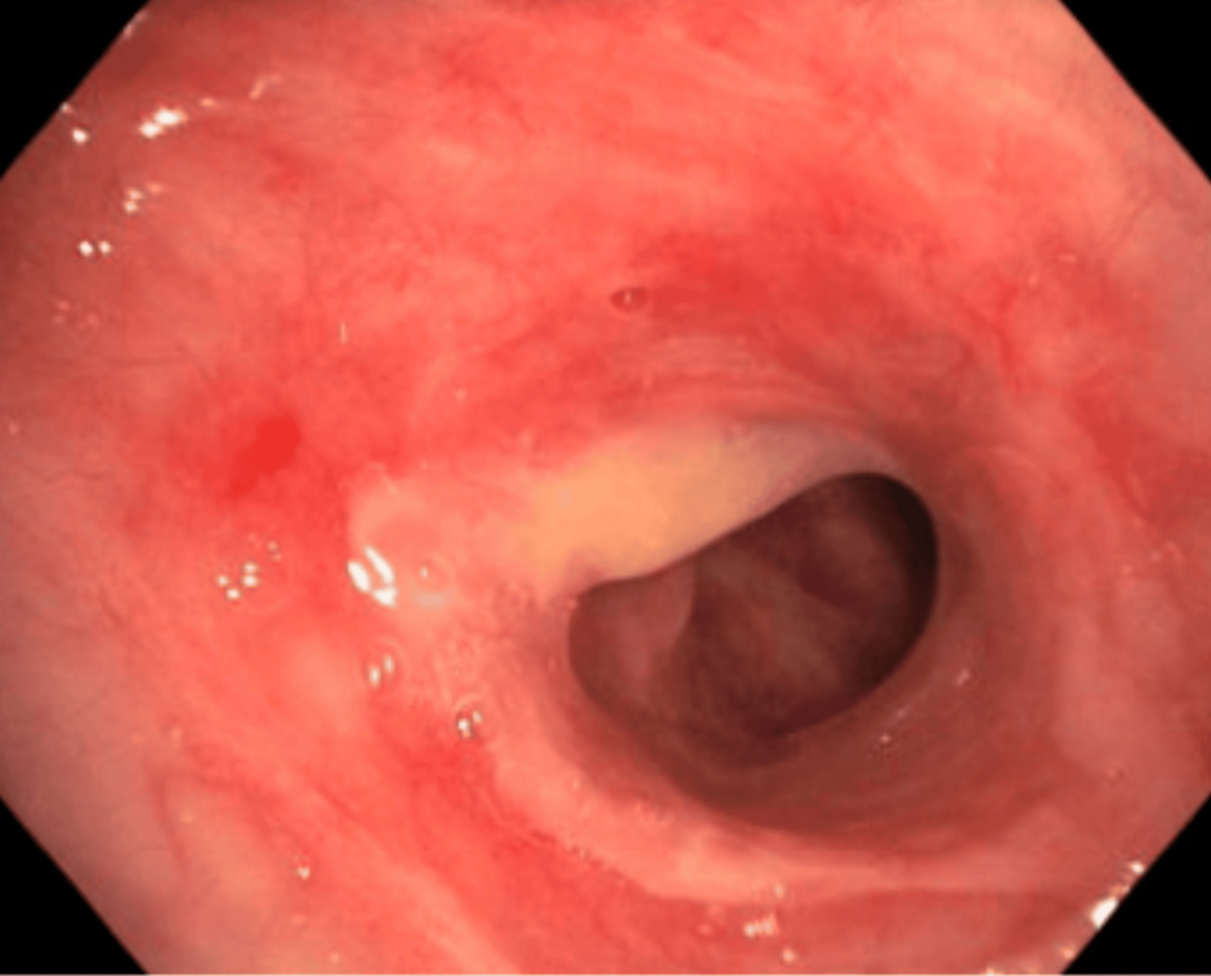
SGS before dilatation visualized during bronchoscopy SGS: subglottic stenosis

The patient then underwent therapeutic bronchoscopy, which revealed a 75%-80% stenosis of the subglottic space (Figure 2). The vocal cords and the remainder of the pulmonary tree were structurally unremarkable.

**Figure 3:**
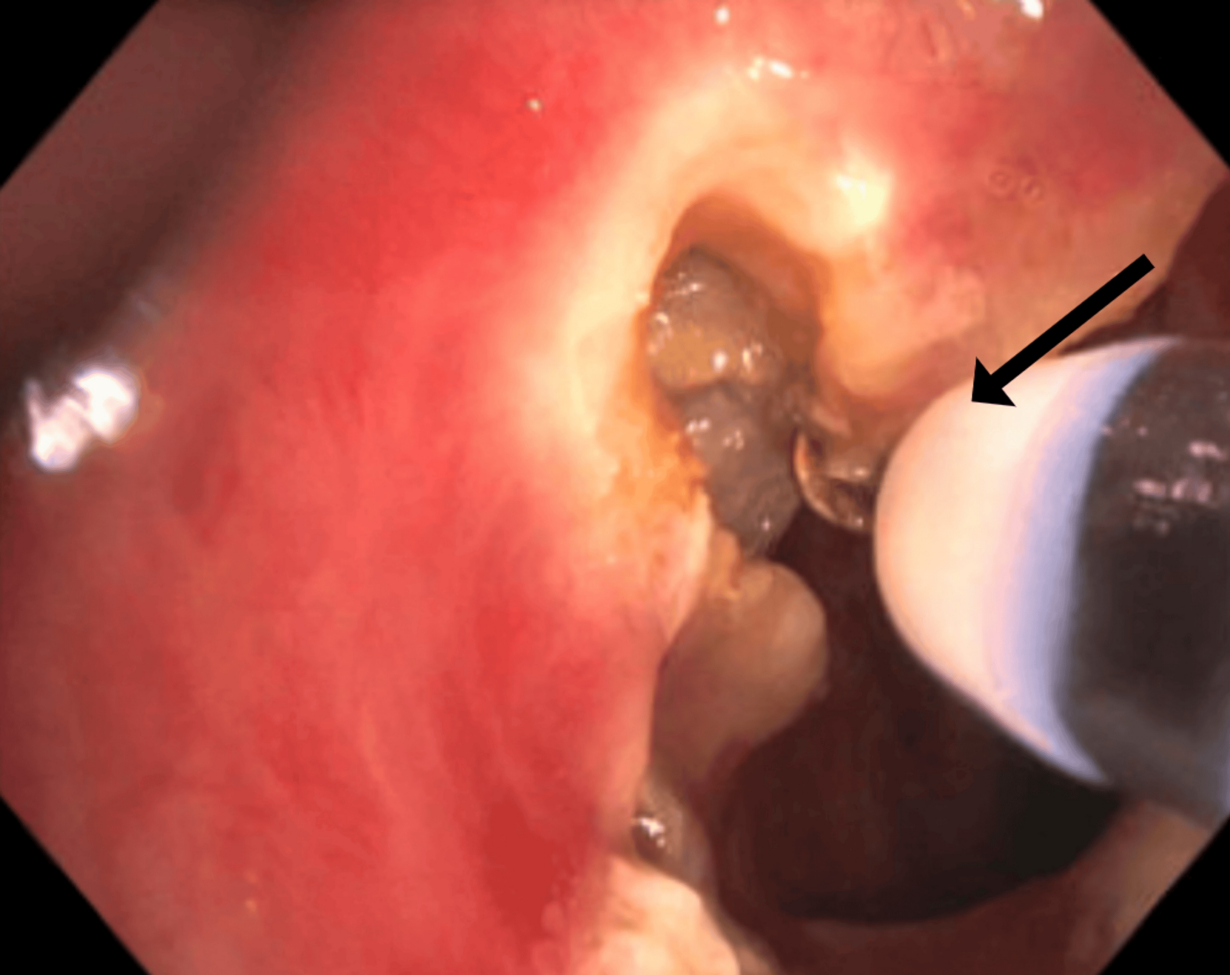
Electrocautery knife cuts of the SGS (black arrow) SGS: subglottic stenosis

**Figure 4:**
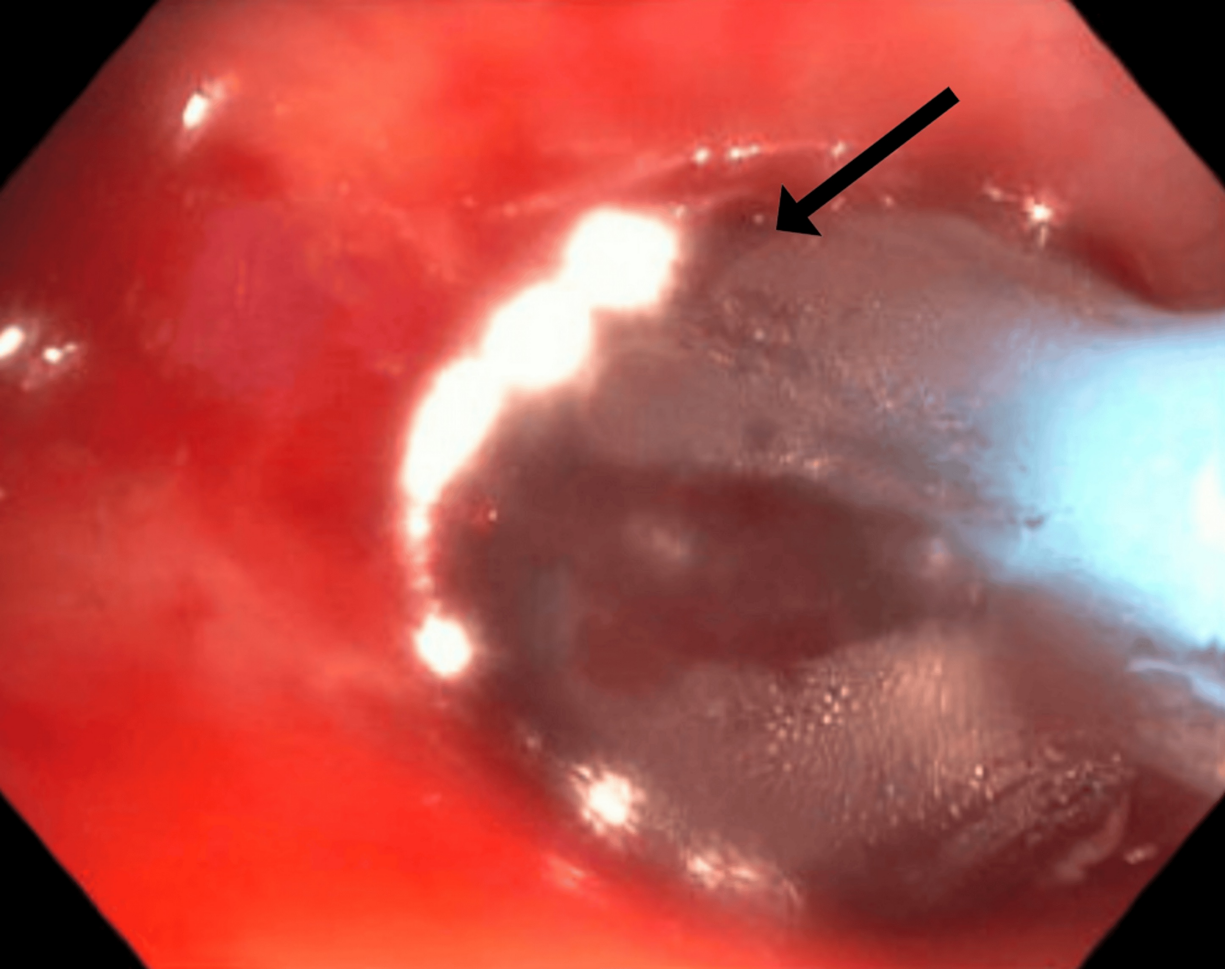
Balloon dilation of the SGS The black arrow points to the balloon dilator SGS: subglottic stenosis

**Figure 5:**
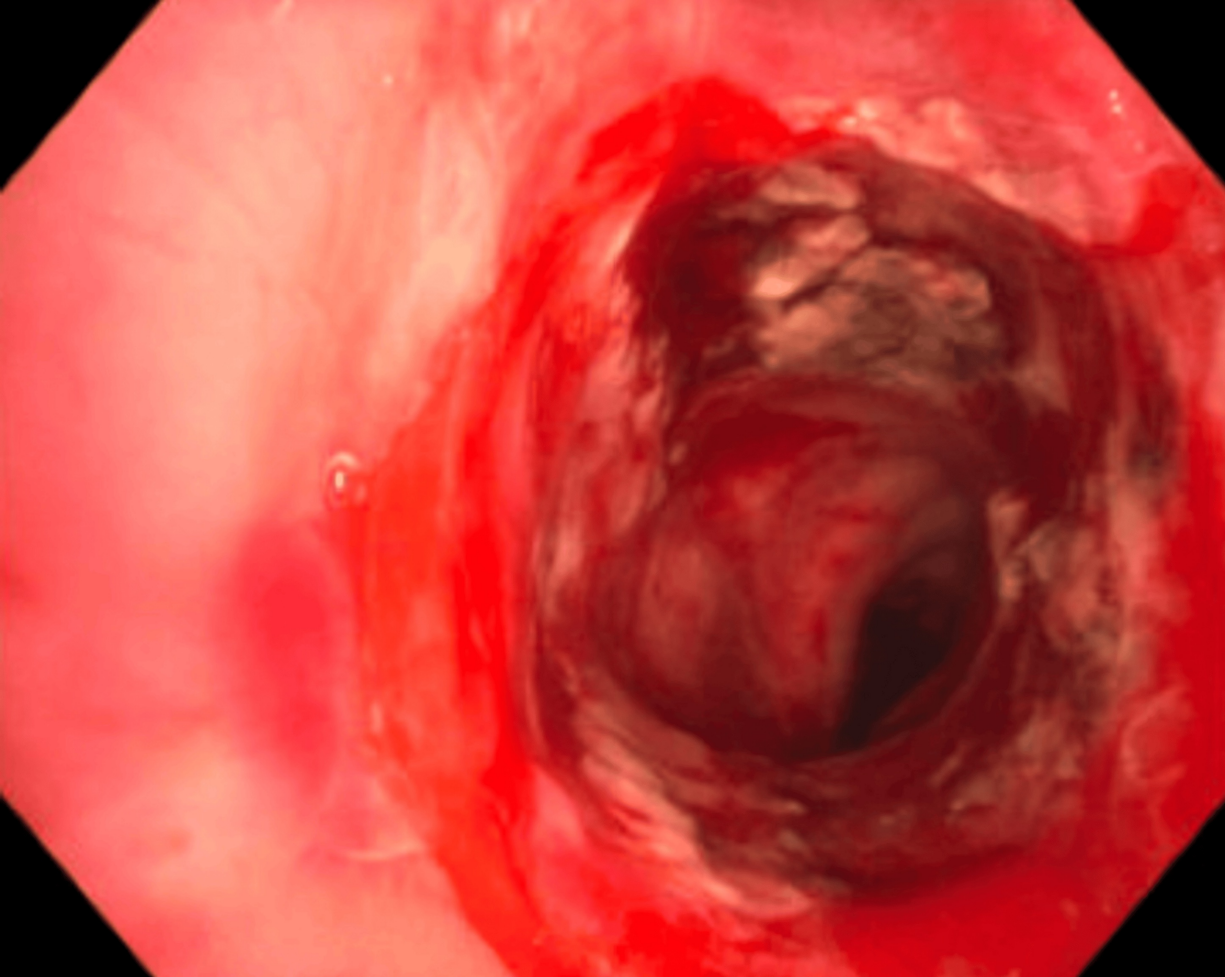
Postdilation view of the SGS with return of lumen to 80% of normal size SGS: subglottic stenosis

Treatment involved electrocautery incisions using the HybridKnife® (T-Type, I-Jet, Erbe Elektromedizin GmbH, Tübingen, Germany) to address the SGS, followed by balloon dilation in the subglottic space using the elation pulmonary balloon dilator (2 cm × 12-13.5-15 mm; Merit Medical, South Jordan, UT) with a controlled radial expansion balloon (Figures 3, 4). The balloon was inflated to 8 atm for one minute and repeated three times. After dilation, the airway lumen was estimated to be approximately 80% of its normal diameter (Figure 5).

## Discussion

The management of recurrent SGS remains challenging due to its high rate of recurrence following intervention. Monitoring for recurrence historically relies on patients reporting their subjective symptoms, such as stridor, dyspnea, and cough. Recent studies emphasize the importance of objective and reliable measures for assessing disease severity and guiding treatment decisions. The subglottic stenosis 6 (SGS-6) questionnaire has emerged as a validated tool for measuring quality of life and tracking disease progression in SGS patients. Studies have demonstrated a significant correlation between SGS-6 scores and pulmonary function test (PFT) results [3]. This emphasizes the potential utility of PEF as a noninvasive, low-cost predictive test in clinical practice to guide the timing of interventions.

Pulmonary function metrics, particularly PEF and the expiratory disproportion index (EDI), have consistently proven to be reliable indicators of disease severity and progression in SGS. A systematic review by Alshareef et al. demonstrated significant improvements in PEF and EDI following intervention, further validating their role in assessing therapeutic outcomes [4]. Similarly, the diagnostic accuracy of spirometry, particularly EDI, has been highlighted as a cost-effective and accessible tool for differentiating SGS from asthma, addressing a common source of diagnostic delays [5].

Despite these advancements, variability in clinical decision-making remains a significant obstacle. A survey of 145 laryngologists revealed substantial heterogeneity in treatment protocols, emphasizing the absence of standardized guidelines [6]. This variability highlights the critical need for consensus-driven approaches to improve patient outcomes and optimize resource utilization.

Emerging technologies, such as mobile phone applications for real-time PEF tracking, show promise in addressing gaps in longitudinal disease monitoring. The findings from Kimura et al. suggest that trends in PEF decline, rather than immediate postoperative improvements, are more predictive of recurrence risk, supporting a shift toward personalized monitoring strategies [7]. These trends align with the findings observed in our patient's case.

Finally, studies have consistently shown an association between earlier otolaryngology referral, imaging, and PFTs with reduced diagnostic delays, further emphasizing the importance of prompt and accurate diagnosis [8]. Improved awareness among primary care providers, along with the integration of standardized spirometry measures into routine evaluations, could significantly reduce the burden of delayed diagnosis and treatment.

After diagnosing SGS in our patient, we implemented the use of peak flow data. The patient was instructed on how to record and share this information through electronic medical records, allowing us to assess the need for intervention. Home monitoring of peak flow provides objective data that complements the patient's reported symptoms, facilitating informed decisions regarding subsequent SGS dilation. This method is user-friendly and significantly more cost-effective than in-lab spirometry and PFTs.

In the case of our patient, it was established that the onset of progressive symptoms, along with a decline in PEF to approximately 250 L/minute, would warrant clinical reassessment to determine the need for subglottic dilation, following the exclusion of alternative etiologies contributing to the symptomatology. It is important to note that this threshold is patient-specific and may vary according to age, sex, and the presence of comorbid conditions [1]. For this reason, it is imperative that both clinicians and patients routinely monitor PEF values before and after intervention. This practice facilitates the development of an individualized baseline and aids in identifying a reproducible threshold associated with symptom onset, thereby guiding timely and appropriate clinical intervention.

## Conclusions

In conclusion, the management of SGS remains challenging due to its high recurrence rate and variability in treatment approaches. This highlights the need for standardized care protocols to guide early and effective intervention. The integration of remote tools, such as PEF monitoring, has proven invaluable in enabling both patients and providers to proactively identify when intervention is needed. Increasing awareness of these advancements and further validating remote technologies can facilitate the development of standardized protocols, ultimately improving patients' quality of life while streamlining clinical workflows.
